# Investigating the *Leishmania donovani sacp* Gene and Its Role in Macrophage Infection and Survival in Mice

**DOI:** 10.3390/tropicalmed7110384

**Published:** 2022-11-18

**Authors:** Kayla Paulini, Patrick Lypaczewski, Wen-Wei Zhang, Dilhan J. Perera, Momar Ndao, Greg Matlashewski

**Affiliations:** 1 Department of Microbiology and Immunology, McGill University, Montreal, QC H3A 2B4, Canada; 2 Division of Experimental Medicine, McGill University, Montreal, QC H4A 3J1, Canada; 3 Infectious Diseases and Immunity in Global Health Program, Research Institute of the McGill University Health Centre, Montreal, QC H4A 3J1, Canada; 4 National Reference Centre for Parasitology, Research Institute of the McGill University Health Centre, Montreal, QC H4A 3J1, Canada

**Keywords:** visceral leishmaniasis, CRISPR-Cas9, whole-genome sequencing, virulence, drug target

## Abstract

The protozoan parasite *Leishmania donovani* is a causative agent of the neglected tropical disease known as visceral leishmaniasis, which can be lethal when untreated. Studying *Leishmania* viru-lence factors is crucial in determining how the parasite causes disease and identifying new targets for treatment. One potential virulence factor is *L. donovani’s* abundantly secreted protein: secreted acid phosphatase (SAcP). Whole-genome analysis revealed that the *sacp* gene was present in three copies in wild type *L. donovani*. Using CRISPR-Cas9 gene editing; we generated a *sacp* gene knockout termed LdΔSAcP, which demonstrated a loss of both the SAcP protein and an associated reduction in secreted acid phosphatase activity. Genome sequencing confirmed the precise dele-tion of the *sacp* gene in LdΔSAcP and identified several changes in the genome. LdΔSAcP demonstrated no significant changes in promastigote proliferation or its ability to infect and survive in macrophages compared to the wildtype strain. LdΔSAcP also demonstrated no change in murine liver infection; however, survival was impaired in the spleen. Taken together these results show that SAcP is not necessary for the survival of promastigotes in culture but may support long-term survival in the spleen. These observations also show that the use of CRISPR gene editing and WGS together are effective to investigate the function and phenotype of complex potential drug targets such as multicopy genes.

## 1. Introduction

The intracellular protozoan parasite *Leishmania* is the causative agent of leishmaniasis and the severity of human leishmaniasis is species specific, ranging from self-healing cutaneous leishmaniasis to the potentially fatal visceral leishmaniasis [1]. There are over 1 million new cases of cutaneous leishmaniasis annually, predominantly in Africa, South America and the Middle-East, and approximately 50,000 new cases of visceral leishmaniasis annually in the Indian Subcontinent, South America and Eastern Africa [1,2]. The *Leishmania* life cycle is dimorphic: the promastigote is a long flagellar cell present in the sand fly vector, and the amastigote is a circular non-flagellar cell present predominantly in the phagolysosome of mammalian host macrophages [3]. In the case of *Leishmania donovani*, the only known mammalian host is the human, and this species causes visceral leishmaniasis, which is fatal if not treated [1]. Patients previously infected with *L*. *donovani* are protected from subsequent infection suggesting that it should be possible to develop a vaccine; however, no safe or effective prophylaxis vaccine is currently available for human leishmaniasis [4]. 

Virulence factors, particularly secreted ones, are good potential targets for the development of vaccines and novel drug treatment approaches [5]. Many *Leishmania* virulence factors have been characterized, such as lypophosphoglycan (LPG), glycoinositolphospholipids (GIPLs), proteophosphoglycan (PPGs), and glycoprotein 63 (GP63) [6]. An example of a well-characterized virulence factor is A2, which is required for parasite survival in visceral organs and is absent in most cutaneous species of *Leishmania* [7]. Although some of these virulence factors could represent drug targets, the specific role of others has yet to be elucidated. One such potential virulence factor is the secreted acid phosphatase (SAcP) that is conserved among all *Leishmania* species and is among the highly abundant secreted proteins in *L. donovani* promastigotes, as shown by pulse labeling total secreted proteins, followed by the immunoprecipitation of SAcP [8,9,10,11]. In vitro studies have determined that SAcP is most enzymatically active in acidic environments and has a wide range of substrates including, but not limited to, glucose 1-phosphate, fructose 6-phosphate and fructose 1,6-diphosphate [12]. The neutralization of SAcP could attenuate the survival of *L. donovani* in infected individuals and thus represent a potential drug target. 

*Leishmania* species are generally considered to be diploid organism although chromosomal aneuploidy is common across all species. The *sacp* genes are located on chromosome 36 and on a circular extrachromosomal episomal DNA in *L. donovani*, and the copy number of this episome appears to vary in different clinical isolates with strains resistant to antimony having a higher copy number [13]. In addition to the *sacp* gene, the episome contains a hypothetical gene and a gene encoding a mitogen-activated protein kinase (MAPK) [13]. It was reported that deletion of all *sacp* genes from chromosome 36 in *Leishmania mexicana* was not associated with a loss of virulence; however, loss of the *mapk* gene located near the *sacp* gene was associated with reduced amastigote fitness in vitro [14] and in mice [15,16]. To our knowledge, the *sacp* gene has never been deleted in *L. donovani* nor has *sacp* been deleted in *Leishmania* spp. without affecting nearby chromosomal genes. We were interested in investigating the *sacp* gene in *L. donovani*, because it is highly secreted and therefore may play an essential role in the life cycle and may represent a drug or antibody target [11,13]. Our previous attempts to delete the *sacp* gene from *L. donovani* using traditional homologous recombination with a selectable marker gene have been unsuccessful, because the *sacp* gene is a multicopy gene where the copy number varies depending on the isolate and because *sacp* is part of a multigene family, which includes the membrane-bound acid phosphatase (MAcP) that shares a conserved domain with SAcP. Previous studies have suggested that MAcP is involved in blocking the production of oxygen metabolites in the phagolysosome of host macrophages and neutrophils, suggesting that MAcP plays a role in protecting the parasite from toxic oxygen metabolites in the host [17,18,19]. 

New genome editing technologies, such as CRISPR-Cas9, have greatly improved our ability to study proteins of interest by specifically deleting or altering the genes encoding them [20]. Our lab has adapted this technology for use in *L. donovani* and has shown that CRISPR-Cas9 gene editing can be applied to multicopy gene deletions [21,22]. Thus, using CRISPR-Cas9 gene editing, we created a knockout strain termed LdΔSAcP (*L. donovani sacp* gene deleted mutant). where all copies of the *sacp* genes, including the episome, were deleted followed by whole-genome sequencing and a phenotypic analysis. The overall objective was to determine the role of the SAcP promastigote viability in culture and amastigote survival in macrophages and infected BALB/c mice. Further, we demonstrate that whole-genome sequencing can be informative to help interpret results with gene-edited mutants. 

## 2. Materials and Methods

### 2.1. Leishmania Strain and Culture Media 

*L. donovani* 1S2D (MHOM/SD/62/1S-Cl2D) was grown at 26 °C in M199 (Sigma, St. Louis, MO, USA, M0393-10X1L) supplemented with 10% heat-inactivated fetal bovine serum (FBS), 40 mM HEPES, 0.1 mM adenine, 5 mg/L hemin, 1 mg/L biotin, 1 mL/L biopterin, 50 U/mL penicillin, and 50 U/mL streptomycin (M199-complete). Parasites were passaged weekly in parallel into fresh media at a 40-fold dilution. 

### 2.2. Whole-Genome Sequencing and Analysis 

DNA was extracted from the parental *L. donovani* 1S2D and the mutant LdΔSAcP strains at identical timepoints using the GeneJet Genomic DNA Purification Kit (Thermo Scientific, Vilnius, Lithuania, K0722) as per the manufacturer’s instructions, with a modified elution buffer consisting of 10 mM Tris-HCl pH 8.0 with 0.1 mM of EDTA. Samples were sent to Centre d’Expertise et de Services Genome Quebec for PCR-free library preparation and sequencing on the Illumina NovaSeq 6000 platform with an S4 cell in PE mode yielding 35 M reads of 150 bp in length. The strategy for strain comparison was previously described [23]. Briefly, fastq files were processed with the Burrows-Wheeler Aligner (BWA) using the command “bwa mem” to create a sam file [24]. The module samtools was used with the command “samtools view” to make an unsorted bamfile followed by “samtools sort” to create a sorted bamfile [25]. The file was then converted to an mpileup using the command “samtools mpileup” [25]. Coverage was determined using the bedtools module with the command “bedtools coverage -mean” for coverage across the whole genome and “bedtools coverage -d” for the coverage per base pair over a gene of interest [26]. Coverage per base pair was graphed using GraphPad Prism 6. SNPs were analyzed using the mutation caller VarScan to create a variant caller format (vcf) file [27]. Using the Galaxy platform [28], SnpEff was used to annotate the variants, and Bedtools Intersect Intervals was used to compare SNPs. Data was inspected using Integrative Genomics Viewer (IGV) Software [29].

Circos plots showing copy number variants were created using the mean coverage files described above and the circos module. The command “circos -conf” was used to generate the circos plot image [30]. 

Additional nanopore sequencing was performed using the parental *L. donovani* 1S2D DNA to confirm the presence and size of the *sacp* gene containing episome. Extracted DNA was sent to the Advanced Genomic Technologies Laboratory at the McGill Genome Center. An Oxford Nanopore Technologies library was prepared using the Native Ligation Kit and sequenced using a PromethION R9.4.1 flow cell on a PromethION/24 instrument. The genomic *sacp* containing locus was assembled de novo using the raw Nanopore reads using the *canu* assembler [31], and the raw reads were then aligned to this new reference as described above. Data was viewed in IGV and in SnapGene. Sequence homology and the sharp change in coverage at either edge were used to determine the boundaries of the potential episome and generate circular sequence for the episome. Individual reads that only mapped to the edges of episome homologous portion were manually selected and realigned to the circular reference corresponding to the ~14 kb episome to determine if they spanned the junction.

### 2.3. Generation and Confirmation of CRISPR-Cas9 Knockouts

The protocol for deletion with CRISPR-Cas9 using two gRNAs was previously described [22,32]. All primers were obtained from Alpha DNA. The gRNAs were designed using the Eukaryotic Pathogen CRISPR guide RNA/DNA Design Tool [33]. The primers LdSAcP1 and LdSAcP2 each containing the gRNA1 and gRNA2, respectively, and the *Bbs* I cut site were used to PCR amplify HDV and hammerhead ribozymes. The insert and pLdCN2 vector were digested with *Bbs* I and ligated together. Many (1 × 10^8^) parasites were transfected with 10 µg of plasmid pLdCN2 containing the gRNAs, then selected using 50–100 µg/mL G418. Primers LdSAcPBleF and LdSAcPBleR2 were used to create the donor DNA by amplifying the bleomycin resistance gene with homology arms to the Cas9 cut sites. Parasites were subsequently transfected with the above-mentioned donor DNA and selected using 50–100 µg/mL of bleomycin. Cells underwent single-cell cloning in 96-well plates and PCR analysis with primers LdSAcPF2 and LdSAcPR of each clone confirmed diploid deletion of the *sacp* genes. All gRNAs and primers used herein are described in Table 1. The knockout strain was passaged in antibiotic-free media to remove the CRISPR plasmid, and the wildtype was passaged in parallel prior to DNA isolation and phenotypic characterization.

### 2.4. SDS PAGE and Western Blot Analysis

Promastigotes (1 × 10^6^) were centrifuged at 2400× *g* for 5 min, and the cell culture supernatant was separated from the pellet by pipetting. The pellet was washed twice in PBS and centrifuged at 2400× *g* for 5 min, then resuspended in diluted 4× loading buffer (4% SDS, 20% glycerol, 0.125 M Tris-HCl, 100 µL/mL beta-2-marcaptolethanol, and 0.004% bromophenol blue). Cell culture supernatant was diluted in 4× loading buffer. Samples were heated at 95 °C for 3 min, then centrifuged at maximum speed for 1 min. Cell culture supernatant and pellet samples were loaded on an 8% acrylamide gel and run at 90 V for 1 h. The gel was transferred to a nitrocellulose membrane using semi-dry transfer at 20 V for 30 min.

Membranes were blocked for 1 h at room temperature in 10% milk in PBS-tween, then blotted overnight at 4 °C with 1/100,000 primary antibody 172-3 (Dwyer lab, NIAID. Rockville, MD, USA). Membranes were washed 5 times for 5 min each with 5% milk in PBS-tween. Anti-rabbit secondary IgG antibody (Abcam, Waltham, MA, USA) was diluted to 1/10,000 and incubated for one hour at room temperature. The previous wash steps were repeated then the membrane was coated with ECL (Zm Scientifique, Montreal, Canada). Membranes were exposed to film for 30 s then developed.

### 2.5. Acid Phosphatase Activity Assay

Acid phosphatase (AP) activity was determined using the Acid Phosphatase Activity Fluorometric Assay Kit (BioVision, Milpitas, CA, USA K421-500). Briefly, 1 × 10^5^ promastigotes were centrifuged at 2400× *g* for 5 min, the cell culture supernatant was removed from the pellet by pipetting. The kit instructions were followed for both the parasite pellet and cell culture supernatant samples. Standard curve was performed as described by the kit. The assay was performed in a black 96-well plate, and the results were read on the PerkinElmer EnSpire 2300 Multilabel Reader with the optimized excitation wavelength of 330 nm and emission wavelength of 440 nm. The final calculations were performed based on the formula for AP activity provided in the kit instructions. Graphs were illustrated using GraphPad Prism 6, and statistical analysis was performed using the two-tailed unpaired Student’s *t*-test.

### 2.6. Promastigote Proliferation Curves

Promastigotes were diluted to 1 × 10^5^ cells/mL in T-25 flasks containing M199-complete and kept in a 26 °C incubator for 7 days. Each day, media-containing parasites was removed from the flask and diluted with 1% formaldehyde. Ten microliters were counted in a cell-counting chamber, and the values were multiplied by the appropriate dilution factors. The data were plotted using GraphPad Prism 6. Statistical analysis was performed using nonlinear regression (curve fit).

### 2.7. In Vitro Macrophage Infections

THP1 monocytes were obtained from the Li-Jessen lab (McGill, Montreal, Canada) and were cultured in RPMI 1640 containing L-glutamine and sodium bicarbonate (Sigma) supplemented with 10% heat inactivated FBS and 1% penicillin–streptomycin at 37 °C in 5% CO_2_. THP1 cells with a viability of 90–95% were seeded on 8-well Nunc^TM^ Lab-Tek^TM^ II Chamber Slides at 5 × 10^5^ cells/well in the media mentioned above supplemented with 50 ng/mL phorbol-12-myristate-13-acetate (PMA). After 24 h, the THP1 monocytes were adherent and deemed macrophages and washed with prewarmed PBS twice then given 400 µL of the complete RMPI 1640 media without PMA. After 3 days in culture, stationary-phase LdWT or LdΔSAcP promastigotes were used to infect the macrophages at 10:1. After 6 h, the wells were washed with prewarmed PBS 4 times. The 6-h time point chamber slides were removed for staining, whereas the other time points were given 400 µL of fresh RPMI 1640 until days 1–4, where the chamber slides were fixed. After 3 days, old media was removed from the remaining chamber slides and replaced with fresh RPMI 1640.

Wells were fixed with methanol containing fast-green for 60 s. Wells were stained with eosin for 30 s then Azur A for 50 s. Once dry, slides were viewed by light microscopy at 100×, and at least 100 macrophages per well were counted. Results were graphed using GraphPad Prism 6, and statistical analysis was done with the two-tailed unpaired Student’s *t*-test at each time point.

### 2.8. BALB/c Mouse Infections

Six- to eight-week-old female BALB/c mice were purchased from Charles River Laboratory (Senneville, QC, Canada) and were infected via tail vein with 1 × 10^8^ promastigotes (LdWT or LdΔSAcP), as previously detailed [34]. After 5 weeks, mice were sacrificed, and liver and spleens were processed for analysis. Livers were weighed, and imprints were made on glass slides with staining performed as described for THP1 cells above. Infection levels were measured by determining the number of parasites per macrophage in 1000 macrophages and multiplying by the weight of the liver (g) to obtain LDU. Spleens were weighed, homogenized and serial diluted in a 96-well plate containing M199-complete media. Infection levels were measured by determining the parasites per well and calculating with the appropriate dilution factor.

## 3. Results

### 3.1. sacp Gene Copy Number in the L. donovani 1S2D Used in This Study

**Figure 1:**
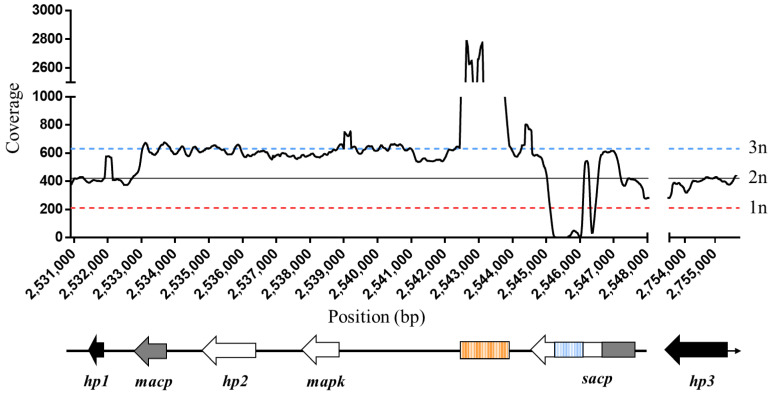
*sacp* gene copy number in the *L. donovani* 1S2D used in this study. The read depth coverage by base pair was determined using the module bedtools coverage. An analysis was performed between positions 2,530,900 and 2,548,000 and between positions 2,753,500 and 2,755,800 of chromosome 36 spanning the following genes: hypothetical protein 1 (*hp1*, LdCL_360075600), the membrane-bound acid phosphatase (*macp*, LdCL_360075700), the hypothetical protein 2 (*hp2*, LdCL_360075800), the mitogen-activated protein kinase (*mapk*, LdCL_360075900), the secreted acid phosphatase (*sacp*, LdCL_360076000) and hypothetical protein 3 (*hp3*, LdCL_360080400). The schematic representation (bottom) shows episomal genes in white and non-episomal genes in black. The portion of *sacp* that is genetically nearly identical to *macp* is shown in grey. The two genetically different regions with repetitive GC-rich sequences are striped orange and striped blue. The coverage graph (top) shows the sliding window read depth coverage as a function of position in base pairs. The average coverage for diploid genes (2n ≈ 400×) is the solid black line, the average coverage for haploid genes (1n ≈ 200×) is the dotted red line and the average coverage for triploid genes (3n ≈ 600×) is the dotted blue line. Note: the axis was split between 2,548,000 and 2,754,000 to condense the cluster of *sacp* genes present in the LdSL-CL reference genome to be more representative of the *L. donovani* 1S2D genome.

The *sacp* gene family is located on the usually diploid chromosome 36, in addition to reportedly being located on an episome that varies in copy number between *L. donovani* strains [13]. Therefore, it was necessary to determine the *sacp* copy number in the *L. donovani* 1S2D lab strain using Illumina whole-genome sequencing (WGS) to guide the gene knockout study design. We generated a section of the genome on chromosome 36 containing a single copy of the *sacp* gene and adjacent genes based on the *L. donovani* CL-SL reference [23] (available for download on https://tritrypdb.org/tritrypdb/app/record/dataset/DSaa86d09f50 (accessed on 20 December 2020)) spanning positions 2,530,900–2,548,000 bp and 2,753,500–2,755,800 bp schematically represented in Figure 1. The Illumina reads were aligned to this reference genome, and the coverage was determined at each base pair position (Figure 1). Since *Leishmania* spp. display a pattern of mosaic aneuploidy and chromosome 31 is known to be tetraploid in all species [35], we used the median coverage of the reads across chromosome 31 in the *L. donovani* CL-SL reference genome (approximately 800×) to define a tetraploid gene, or 4n, to be equal to 800×. From this value, we inferred that 1n = 200× (haploid), 2n = 400× (diploid) and 3n = 600× (triploid) to determine the gene’s copy number. The hypothetical genes located upstream (*hp1* = LdCL_360075600) and downstream (*hp3* = LdCL_360080400) of the *macp* and *sacp* genes are diploid. The hypothetical gene 2 (*hp2* = LdCL_360075800) and the *mapk* gene (LdCL_360075900) are triploid. Overall, the read depths indicate that there are 3 copies of the region spanning 2,533,000–254,800, consistent with two copies of this region from the diploid chromosome 36 and the third copy from one episome, as previously reported [13].

The *sacp* and *macp* genes are complex to analyze due to their genetic similarities and the presence of short repetitive GC sequences in the *sacp* gene. Since the first ~900 bp of the *sacp* gene are almost identical to the sequence of the *macp* gene and the Illumina reads are ~150 bp long, the reads are aligned to either the *sacp* or *macp* reference gene. Therefore, it is likely that the Burrows-Wheeler Aligner is aligning reads from the homologous region of *sacp* to the *macp* reference gene. Additionally, the *sacp* specific 5′ sequence is primarily made up of short, GC-rich repetitive sequences (Figure 1, blue striped box) that prevent accurate alignment, which appear as a lack of coverage at positions 2,546,249–2,546,502 in the *sacp* gene. This poor coverage quality over the *sacp* gene region has been previously reported [23]. The intergenic sequence upstream of *sacp* has a coverage of over 2500× (Figure 1, orange striped box). This is a highly repetitive region with the sequence occurring in the intergenic DNA of every chromosome, which explains the sharp peak at positions 2,542,536–2,543,809. Considering the reads that are unique to the *sacp* gene with no GC-rich repeats (2,546,618–2,547,089), the average gene coverage is 3n. Taken together, this sequence analysis is consistent with the presence of 3 copies of genes *mapk, hp2* and *sacp* in which two copies are on chromosome 36 and one copy is on an episome.

To confirm the presence of the episome, we performed long-read sequencing using Nanopore sequencing. As shown in Supplementary Figure S1a, a sharp increase in coverage was seen in the SAcP containing genomic locus similarly to that shown in Figure 1. As highlighted by the episome arrows, several reads were found to map to the proposed episome location and stopped abruptly at the edges as they did not align with the flanking genomic sequences. As shown in Supplementary Figure S1b, when these same reads were aligned to a circular reference corresponding only the episome sequence, the alignments looped around the other end of the sequence, confirming its circular nature.

### 3.2. Generation of the L. donovani Mutant, LdΔSAcP, Using CRISPR-Cas9 Gene Editing

**Figure 2:**
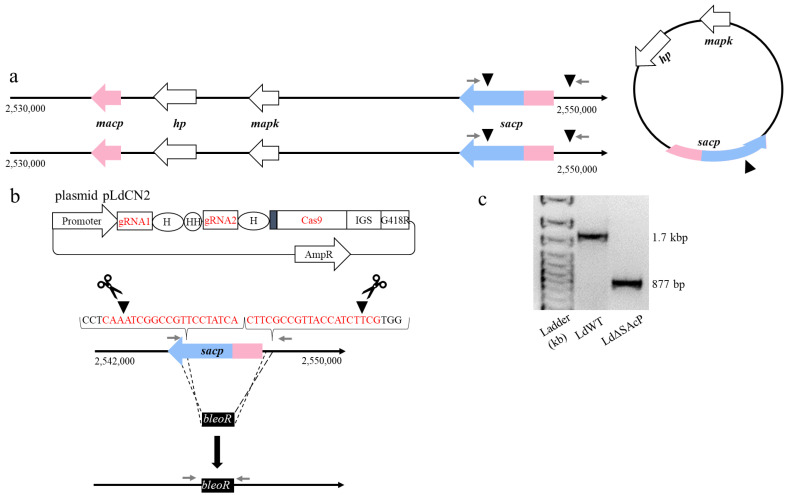
Generation of the *L. donovani* mutant, LdΔSAcP, using CRISPR-Cas9 gene editing. (**a**) Genetic representation of the *sacp* loci. The usually diploid chromosome 36 is illustrated from position 2,530,000 to position 2,550,000 containing the *macp* gene (LdCL_360075700) in pink, the *hp* gene (LdCL_360075800) in white, the *mapk* gene (LdCL_360075900) in white and the *sacp* gene (LdCL_360076000) in blue, with the homologous sequence to the *macp* gene in pink. The *hp* gene, the *mapk* gene and the *sacp* gene are also encoded on one episome. The black triangles indicate the locations of the Cas9 endonuclease cut sites and the grey arrows represent the primers used in PCR. (**b**) Gene editing strategy to remove *sacp.* Promastigotes were transfected with the CRISPR vector pLdCN2 [22] containing two guide RNAs specific to the *sacp* gene but not to the *macp* gene. The gRNA sequences are in red, and the PAM sequence is in black with the cut sites indicated by the black arrows. Promastigotes were subsequently transfected with a bleomycin resistance gene (*bleoR*), and through microhomology end joining, the *bleoR* gene is integrated into the *sacp* gene. (**c**) Gel-electrophoresis of the PCR analysis with the primers LdSAcPF2 and LdSAcPR (Table S1) of the *sacp* gene from LdWT DNA (1685 bp) and the LdΔSAcP DNA (877 bp).

After determining that there are three copies of the *sacp* gene in this *L. donovani* 1S2D strain, we set out to delete all copies. A schematic representation of the *sacp* gene locus on chromosome 36 and the episome containing the *sacp, mapk and hp2* genes are depicted in Figure 2a. A CRISPR-Cas9 gene editing protocol for *Leishmania* was previously successful in deleting the A2 multicopy gene in *L. donovani* [22], thus we attempted a similar approach to delete the *sacp* genes as illustrated in Figure 2b. *L. donovani* 1S2D promastigotes were transfected with the pLdCN2 plasmid expressing two gRNAs and Cas9: the gRNA1 target is located within the *sacp* gene, and the gRNA2 target is located in the 5′ intergenic region the *sacp* gene (Figure 2a). Promastigotes selected for resistance to G418 were subsequently transfected with a single-stranded donor DNA encoding the bleomycin resistance gene (*bleoR*) flanked by 25 bp homologous sequences to the cut sites of the gRNAs. Successful *sacp* chromosomal gene deletions and replacement with the *bleoR* gene were initially confirmed by PCR (Figure 2c). This PCR reaction however does not formally confirm the episome is removed since it may lack the downstream primer site, although the episome should have been targeted by the internal gRNA and Cas9. We therefore next examined the SAcP protein levels and enzyme activity.

**Figure 3:**
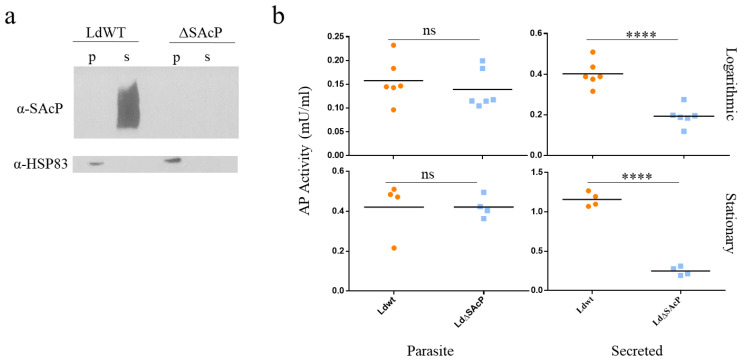
Confirmation that the SAcP protein is lost in LdΔSAcP. (**a**) Western blot analysis of SAcP in *L. donovani 1S2D* wild type (LdWT) and *L. donovani* mutant (LdΔSAcP) strains. Cell lysates (p = parasite-contained proteins) and cell-culture supernatants (s = secreted proteins) were Western blotted using the α-SAcP primary rabbit antibody. No remaining SAcP is detected in the LdΔSAcP strain. HSP83 was used as a loading control for the cell lysate. Full, uncropped image available in Figure S2. (**b**) Acid phosphatase activity was measured in the parasite-contained proteins and in the secreted proteins in the *L. donovani 1S2D* wild type (shown in orange) and mutant parasites (shown in blue) during logarithmic (*n* = 6, parasite *p*-value = 0.4749, secreted *p*-value = 9.383 × 10^−5^) and stationary phases of growth (*n* = 4, parasite *p*-value = 0.9926, secreted *p*-value = 2.435 × 10^−6^), ns > 0.05, **** *p* < 0.0001.

To determine whether the *sacp* gene-targeted mutants lost the expression of SAcP protein, a Western blot analysis was performed using a polyclonal antiserum previously shown to react against SAcP but not MAcP [10]. As shown in Figure 3a, SAcP was detected in the promastigote supernatant but not the pellet derived from wildtype *L. donovani* 1S2D as expected for a secreted enzyme. In contrast, there was no detectable SAcP in the supernatant derived from the LdΔSAcP mutant. Next, the promastigote’s ability to hydrolyze the phosphatase substrate 4-methylumbelliferyl phosphate (MUP) was compared as a measurement of acid phosphatase activity in logarithmic and stationary phases of promastigote proliferation. As shown in Figure 3b, the acid phosphatase activity was lower in LdΔSAcP than in LdWT for the cell culture supernatant proteins but was the same in the pellet containing the parasite-bound proteins. Taken together, the PCR (Figure 2c), the Western blot (Figure 3a) and the enzyme activity assay (Figure 3b) data provide strong evidence that the *sacp* genes have been successfully deleted in the *L. donovani* LdΔSAcP mutant. We attempted to generate a *sacp* gene addback prior to undertaking phenotypic studies on the LdΔSAcP mutant. However, due to the extensive GC repeat sequences present in the *sacp* gene, it was not possible to construct a plasmid vector capable of expressing the *sacp* gene. We therefore performed a whole-genome sequence (WGS) analysis on the LdΔSAcP mutant and the parental wildtype *L. donovani* 1S2D (LdWT) to establish whether the *sacp* gene was specifically deleted and to determine whether there were any differences in their genomes in addition to the gene-edited region in LdΔSAcP.

### 3.3. Whole-Genome Sequencing Shows Specificity of CRISPR-Cas9 Gene Knockout

**Figure 4:**
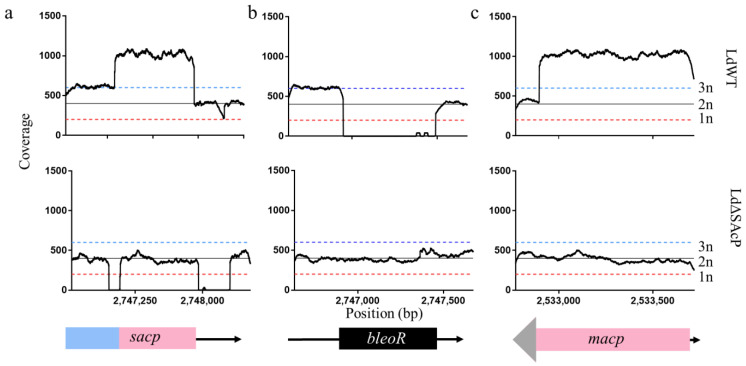
Whole-genome sequencing shows specificity of CRISPR-Cas9 gene knockout. Read depth coverage by base pair was determined using the module bedtools coverage over three separate reference files illustrated at the bottom of the figure. (**a**) The *L. donovani 1S2D* wild type genome (top) and the mutant genome (middle) are aligned to the wild type *sacp* gene (bottom). The *sacp* gene is blue with pink demonstrating the region with genomic similarities between the *macp* gene and the *sacp* gene from 2,747,080 to 2,747,953, which has a coverage of ~1000× in LdWT. Positions 2,748,288–2,748,310 and 2,746,950–2,746,972 are the positions of the gRNAs used to target the *sacp* gene and both positions have an average coverage of 0 in LdΔSAcP. (**b**) The *L. donovani* wild type genome (top) and the mutant genome (middle) are aligned to the *bleoR* gene (black), which was inserted into the *sacp* gene (bottom). LdWT has an average coverage of 0 over the *bleoR* gene whereas LdΔSAcP has continuous coverage. (**c**) The *L. donovani* wild type genome (top) and mutant genome (middle) are aligned to the *macp* reference genome, which is pink (bottom). LdWT has an average coverage ~1000× where the *macp* gene and the *sacp* gene are nearly identical, which drops to ~400× in the LdΔSAcP genome. The red dotted line represents the expected coverage for 1n, the solid black line represents the expected coverage for 2n and the blue dotted line represents the expected coverage for 3n.

WGS of LdΔSAcP and wild type *L. donovani* 1S2D (LdWT) was carried out as described in the methods, and the Illumina reads were aligned to the reference genome *L. donovani* CL-SL [23]. When analyzing the *sacp* gene from sequence positions 2,746,541–2,748,530 on chromosome 36, a drop in coverage at the precise location of gRNA1 and gRNA2 confirmed the deletion of sequences flanking the *sacp* gene in LdSΔAcP compared to LdWT (Figure 4a). Note, a portion of the *macp* gene aligns to the *sacp* gene map due to their genetic similarity, and therefore, the *sacp* gene coverage is greater than the average diploid coverage (400×) and the average triploid coverage (600×) in the LdWT reads and does not drop to zero in the corresponding sequence in LdΔSAcP between 2,747,080 and 2,747,953. In consequence, LdΔSAcP will not have a gene coverage of 0, where the *macp* reads align to the *sacp* portion of the reference genome. The reverse is also true where the *sacp* gene reads align to the *macp* reference genome, resulting in the *macp* genome appearing to have a coverage of 5n, where two copies are from the diploid *macp* gene, two copies are from the chromosomal *sacp* gene and one copy is from the episomal *sacp* gene (Figure 4c, top). When the *sacp* gene is deleted, the coverage graph shows an average diploid coverage over the entire *macp* gene (Figure 4c, middle). Indeed, *macp* remains intact in LdΔSAcP (Figure 4c), demonstrating the specificity of CRISPR-Cas9 gene targeting to the *sacp* genes in the chromosome.

As an additional analysis, an artificial reference genome was generated with the *bleoR* gene in place of the *sacp* gene. When the LdWT genome is aligned to an artificial reference genome with *bleoR* in the place of *sacp*, an average coverage of 0 confirms the absence of *bleoR* in LdWT (Figure 4b). However, when the LdΔSAcP genome containing the integrated *bleoR* gene is aligned to the same artificial reference genome, constant coverage over *bleoR* illustrates the correct integration of *bleoR* in the *sacp* gene in LdΔSAcP (Figure 4b). Taken together, the read coverage further confirms the precise deletion of *sacp* and the integration of *bleoR* on chromosome 36 in LdΔSAcP without affecting the *macp* gene.

**Figure 5:**
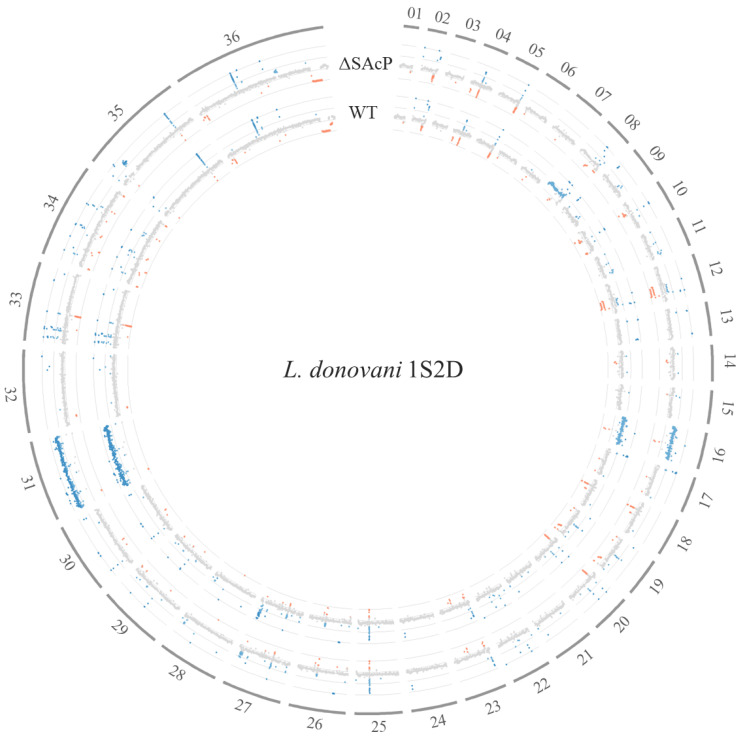
*L. donovani* wild type and mutant whole-genome chromosome analysis. *L. donovani* 1S2D wild type and *L. donovani* mutant LdΔSAcP genomes were sequenced by Illumina, and the reads were aligned to the *L. donovani* CL-SL reference genome [23], and their coverages were analyzed using the module bedtools coverage. The mean coverage for each gene is plotted along the reference genome from chromosome 1 to 36, and LdΔSAcP coverage is normalized to LdWT coverage. Mean coverage is grey, increased copy numbers is blue and decreased copy numbers is red. The inner circle is the *L. donovani* wild type (WT) and the outer circle is the mutant LdΔSAcP genome. The average coverage across the entire genome serves as the baseline for diploid genes. Chromosome 35 contains an amplicon and chromosome 8 has reverted to diploid in the LdΔSAcP genome.

We next examined the whole genome for potential differences between the LdWT and LdΔSAcP genomes with respect to read depths. *Leishmania* are known to utilize chromosome aneuploidy as a rapid way to regulate gene expression [36,37,38]. As shown in Figure 5, LdWT and LdΔSAcP both had four copies of chromosome 31 similar to all *Leishmania* species. In addition, LdWT and LdΔSAcP each had three copies of chromosome 16. It appears, however, that chromosome 8 in LdΔSAcP was the normal diploid compared to LdWT, which was triploid. Second, an amplicon of approximately 89.5 kbp located on chromosome 35 was present in LdΔSAcP but not LdWT. Further, there were five homozygous nonsynonymous SNP differences outside of the CRISPR-edited region in LdΔSAcP on chromosome 36 that were not present in the LdWT (Table S1). The predicted gene products of these genes do not appear to be associated with the activity of SAcP. Although chromosome ploidy and amplicon changes are relatively common in cultured *Leishmania*, it was interesting that there were homozygous SNPs in the same chromosome 36 that was edited, although the SNPs were outside of the edited region.

### 3.4. Comparison of LdWT and LdΔSAcP Promastigotes in Culture and Amastigotes in Macrophages and BALB/c Mice

**Figure 6:**
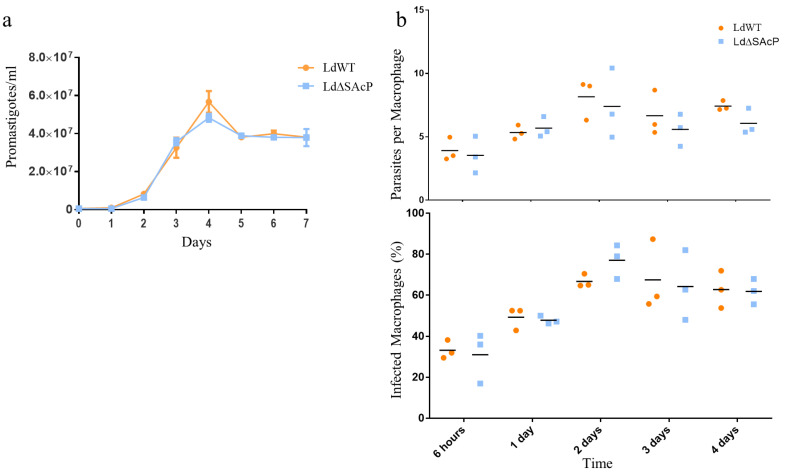
Loss of SAcP does not significantly impact *L. donovani* promastigote or amastigote fitness in vitro. (**a**) *L. donovani* wild type and mutant LdΔSAcP parasites were grown in liquid M199 culture, and proliferation was assessed. The log of promastigotes per milliliter is shown as mean and standard deviation. A curve fit for nonlinear regression was performed. *n* = 3 and *p*-value = 0.9001. (**b**) In vitro THP1 macrophage infection was assessed by the number of parasites per macrophage and the percentage of infected macrophages. Three individual data points and their means are represented on the graph with no significance at each time point based on the two-tailed unpaired Student’s *t*-test.

Promastigote proliferation was determined using an in vitro growth curve comparing LdΔSAcP to LdWT parasites. As observed in Figure 6a, promastigote proliferation in culture was very similar in LdΔSAcP and LdWT parasites. We next assessed amastigote infection and survival in macrophages. THP1 monocytes were differentiated into macrophages with PMA and were infected with LdWT or LdΔSAcP. The level of infection was determined by the number of parasites per infected macrophage and by the percentage of infected macrophages as shown in Figure 6b. Both the initial infection levels and subsequent proliferation of amastigotes over 4 days was not significantly different between LdWT and LdΔSAcP.

**Figure 7:**
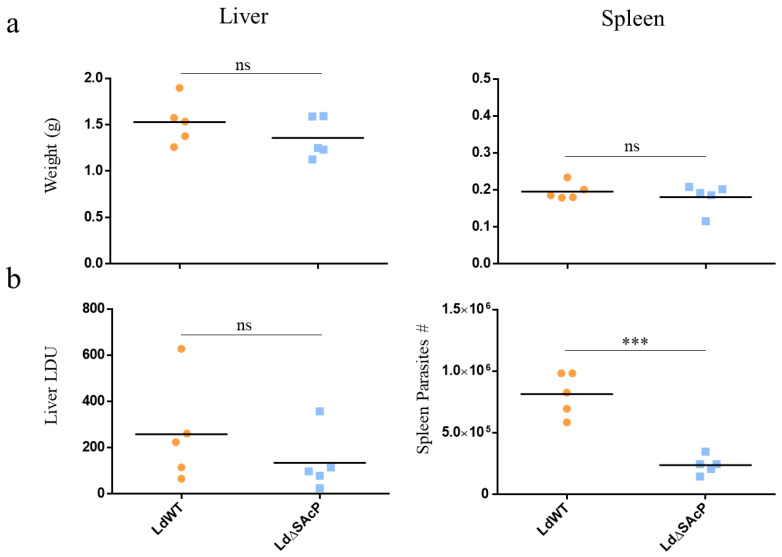
Comparison of LdWT and LdΔSAcP parasites in a mouse model. BALB/c mice were infected with 1 × 10^8^ parasites (LdWT = orange and LdΔSAcP = blue) via teil vein infection. After 5 weeks, mice were sacrificed, and livers and spleens were kept for analysis. (**a**) Livers (left) and spleens (right) were weighed, and the mass was plotted in grams. Two-tailed unpaired Student’s *t*-test was performed (*n* = 5, liver *p*-value = 0.2741, spleen *p*-value = 0.4682). (**b**) Liver LDU was calculating by determining the number of amastigotes per 1000 macrophages and multiplying by mass (g) in the liver imprints. Two-tailed unpaired Student’s *t*-test was performed (*n* = 5, *p*-value = 0.3167). The number of spleen parasites were determined by serial dilution. Two-tailed unpaired Student’s *t*-test was performed (*n* = 5, *p*-value = 0.0008), ns > 0.05, *** *p* < 0.001.

We next compared the ability of LdWT and LdΔSAcP to survive in vivo in the liver and spleen in a BALB/c mouse model. There was no significant difference in the weight of the livers or spleens between LdWT or LdΔSAcP at week 5 following infection via the tail vein (Figure 7a). Although there was no significant difference in the liver LDUs between LdWT and LdΔSAcP, there is a trend suggesting a decreased survival of amastigotes in the liver of the LdΔSAcP infected mice compared to the LdWT-infected mice (Figure 7b, Liver). There was, however, a significant decrease in the number of amastigotes in the spleen of the LdΔSAcP infected mice compared to the LdWT infected mice (Figure 7b, Spleen).

## 4. Discussion

The objective of this study was to examine the role of SAcP in *L. donovani* infection and as a potential drug target. Previous attempts to delete the *sacp* genes from *L. donovani* to make an LdΔSAcP using traditional homologous recombination knockout techniques were unsuccessful, presumably due to the episome copy of *sacp*. Herein, we demonstrated that it was possible to delete the *sacp* gene on the often-diploid chromosome 36 and on the episome by using CRISPR-Cas9 methodology. Loss of SAcP protein in the LdΔSAcP mutant was confirmed by Western blotting and an enzyme assay showing the loss of secreted phosphatase activity. There was, however, no loss of fitness in the LdΔSAcP promastigote proliferation in culture or in vitro infection in macrophages. In BALB/c mice, however, there was a detectable reduction in the infection in the spleen, although liver infections were not significantly different between the LdΔSAcP and LdWT. In the BALB/c mouse model, *L. donovani* initially infects the liver, where it is cleared, before establishing in the spleen [39]. This suggests that LdΔSAcP may have been compromised for the longer-term survival in the spleen but not the initial infection of macrophages in the liver. These findings are somewhat similar to those previously reported, as showing SAcP in *L. mexicana* is not required for infection in mice [15,16], arguing that, similar to in *L. mexicana,* SAcP in *L. donovani* is not a major determinant of virulence. Overall, the loss of SAcP from *L. donovani* 1S2D did not impair promastigote proliferation, infection of macrophages in vitro or infection of the liver suggesting that the phenotypic difference between LdΔSAcP compared to LdWT *L. donovani* 1S2D is relatively minor but potentially contributes to the long-term survival in the spleen. Nevertheless, these data would argue that SAcP may not be a highly effective drug target, or gene target for deletion to make a live attenuated vaccine strain.

A major drawback of this study was the inability to generate an addback plasmid expressing SAcP in the LdΔSAcP mutant. As a result, we sequenced the genome of LdΔSAcP and the parental LdWT to establish the precise CRISPR-targeted deletion of the *sacp* genes and whether there were any other differences in their genomes that could potentially contribute to differences in phenotype between LdΔSAcP and LdWT. The sequence analysis did confirm the correct targeted deletion of the *sacp* genes from chromosome 36. which was supported by the SAcP Western blot and the phosphatase enzyme assay analysis. However, unexpectantly, there were five homozygous non-synonymous SNP differences in LdΔSAcP on chromosome 36 that were not present in the LdWT sequence. It appears that none of the genes containing these SNPs are related to phosphatases or phosphatase targets (Table S1). Additionally, there was an 89.5-kb amplicon on chromosome 35 that was absent in LdWT and chromosome 8 was diploid in LdΔSAcP but triploid in LdWT. Consequently, it is not possible to formally rule out the possibility that the reduced level of infection in the spleen was also due in part to the differences in SNPs, the amplicon and the chromosome 8 aneuploidy. However, any genetic differences between LdΔSAcP and LdWT outside of the CRISPR-targeted region occurred during the in vitro culture of promastigotes. These changes were not associated with any change in promastigote growth characteristics in culture, nor would these changes result from pressure to survive as amastigotes; thus, they are most likely to be random changes. It would be interesting to sequence multiple clones of LdΔSAcP and perform phenotypic experiments on all clones to definitively determine the role of each genetic change seen in LdΔSAcP however, this would not be feasible. Now that WGS has become more accessible, it will continue to be an important analysis in future such studies.

It has been reported that SAcP is more active in promastigotes than in amastigotes [40]. It is possible that SAcP is required for survival of promastigotes in the sand fly. SAcP is more active in acidic conditions [12], and although the sandfly midgut is generally viewed as a basic environment, metacyclogenesis in the sand fly has been reported to occur at low pH and nutrient depletion [41]. It is therefore possible that SAcP plays a role in the later stages of infective metacyclic promastigotes. Future investigations involve studying the implications of SAcP in parasite survival within the vector and the ability of LdΔSAcP to transmit from vector to host are required. Furthermore, it is interesting that the episome encoding the *sacp* gene was upregulated in antimony-resistant clinical isolates of *L. donovani* [13], suggesting a link between antimony and the genes that are present on the episome, including *sacp*, which warrants future investigation.

In summary, evidence is provided for the presence of three *sacp* genes in *L. donovani* 1S2D that were successfully targeted using CRISPR-Cas9. The resulting LdΔSAcP mutant showed a modest change in phenotype, notably a reduced survival in the spleen of infected mice. Whole-genome sequencing revealed that genetic changes outside the CRISPR edited region could also contribute to this phenotype and WGS should become widely used following gene editing.

## Figures and Tables

**Table 1:** Primers and associated sequences.

Primer Name	Primer Sequence
LdSAcP1	5′ ATCGAAGACCTTTGTCTTCGCCGTTACCATCTTCGGTTTTAGAGCTAGAAATAGCAAG
LdSAcP2	5′ ATCGAAGACCCAAACCAAATCGGCCGTTCCTATCACCATGACGAGCTTACTC
LdSAcPBleF	5′ TCTGCGTCCCACCGGAATCCCTCAAGATCTTCATCGGATCGGGTA
LdSAcPBleR	5′ GCGGTGTCCTGGAGCAGCTCCACGAGTCGGTCAGTCCTGCTCCT
LdSAcPF2	5’ CCGCCCACTCAACGATTAT
LdSAcPR	5′ CTGTACTGAGCCTGCGTCAT

## Data Availability

Publicly available datasets were analyzed in this study. This data can be found under the BioProject: PRJNA873111 accession number.

## Supplementary Materials

The following supporting information can be downloaded at: https://www.mdpi.com/article/10.3390/tropicalmed7110384/s1: Figure S1: Nanopore data of the *L. donovani* WT episome Figure S2: Western blot of SAcP in LdWT and LdΔSAcP. Table S1: Non-synonymous SNPs in the LdΔSAcP Genome.
