# Supplementary material for: Investigating the Leishmania donovani sacp Gene and Its Role in Macrophage Infection and Survival in Mice
Source: Trop Med Infect Dis. 2022 Nov 18;7(11):384. doi: 10.3390/tropicalmed7110384 (PMC9694005; doi:10.3390/tropicalmed7110384)
Supplement: Supplementary file 1 [file tropicalmed-07-00384-s001.zip › tropicalmed-1908947-supplementary.pdf]

Table S1. Non-synonymous SNPs in the LdΔSAcP Genome.

Gene IDC	Gene Name	SNP
LdCL_360081200	Acyl-CoA binding protein	Pro257Ala
LdCL_360081300	y113g7b.23 protein-like protein	Val43Ala
LdCL_360081500	Sperm tail/Sperm tail C-terminal domain containing protein	Arg429His
LdCL_360081900	Hypothetical protein	Val6Met
LdCL_360082200	2,3-bisphosphoglycerate-independent phosphoglycerate mutase	Lys481Arg

VarScan and Galaxy's SnpEff Eff were used to call and annotate SNPs respectively. The five non-synonymous homozygous SNPs are listed here. All SNPs are from chromosome 36, have a genotype quality of 255, a high median coverage compared to the median coverage of the entire genome and a read frequency of at least 94%.

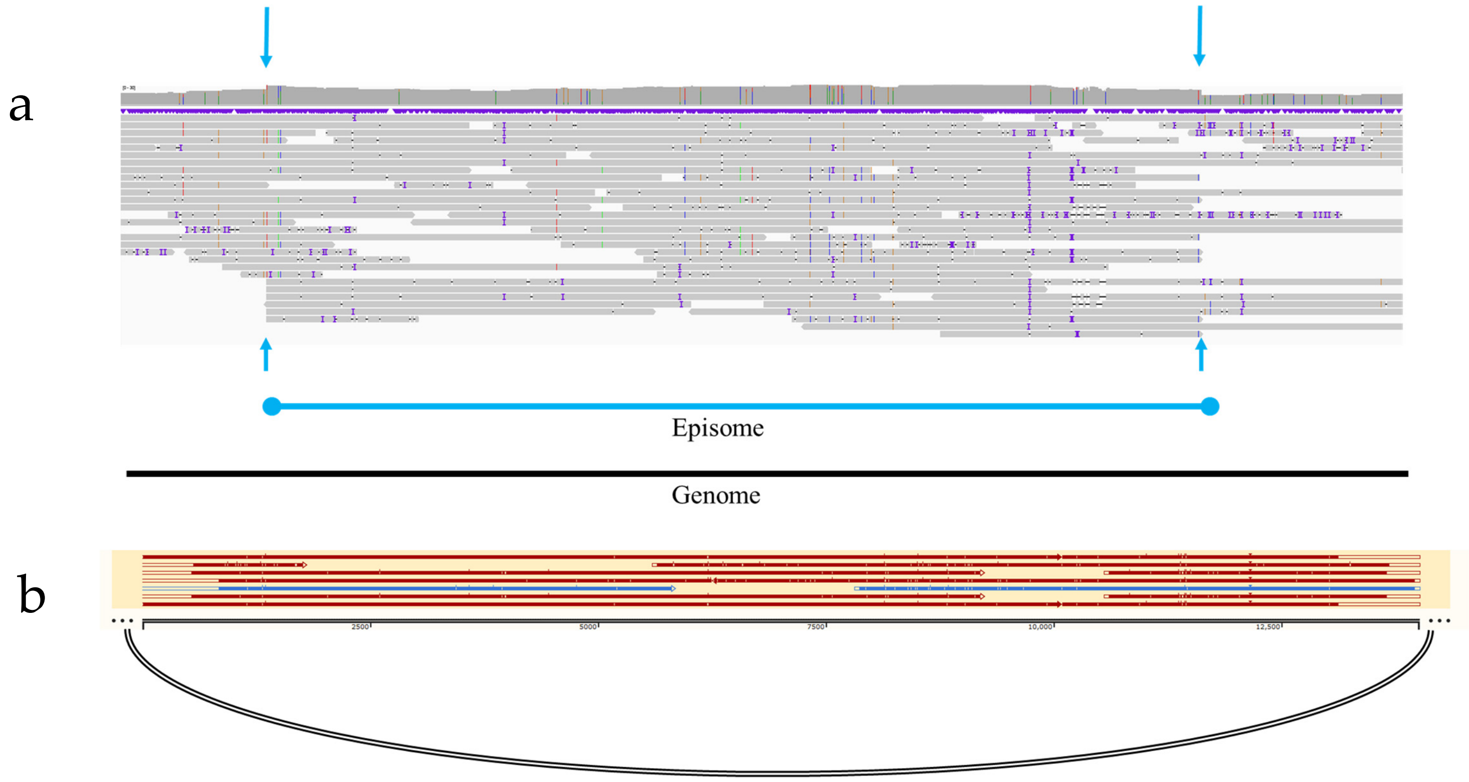

Figure S1. Nanopore data of the *Leishmania donovani* WT episome.

L. donovani nanopore sequencing data was viewed in (a) IGV and (b) SnapGene. The sequence of the episomal DNA should be present within chromosome 36 and as extrachromosomal circular DNA. (a) When the chromosomal reference DNA is linear, a sharp increase followed by a decrease in coverage (indicated by the blue arrows) and sharp end to some of the reads suggests the presence of an episome at the location indicated in blue labeled episome. (b) When the episome reference file is circularized (black), the reads wrap around the episome sequence. Note: each line represents a unique and continuous nanopore read.

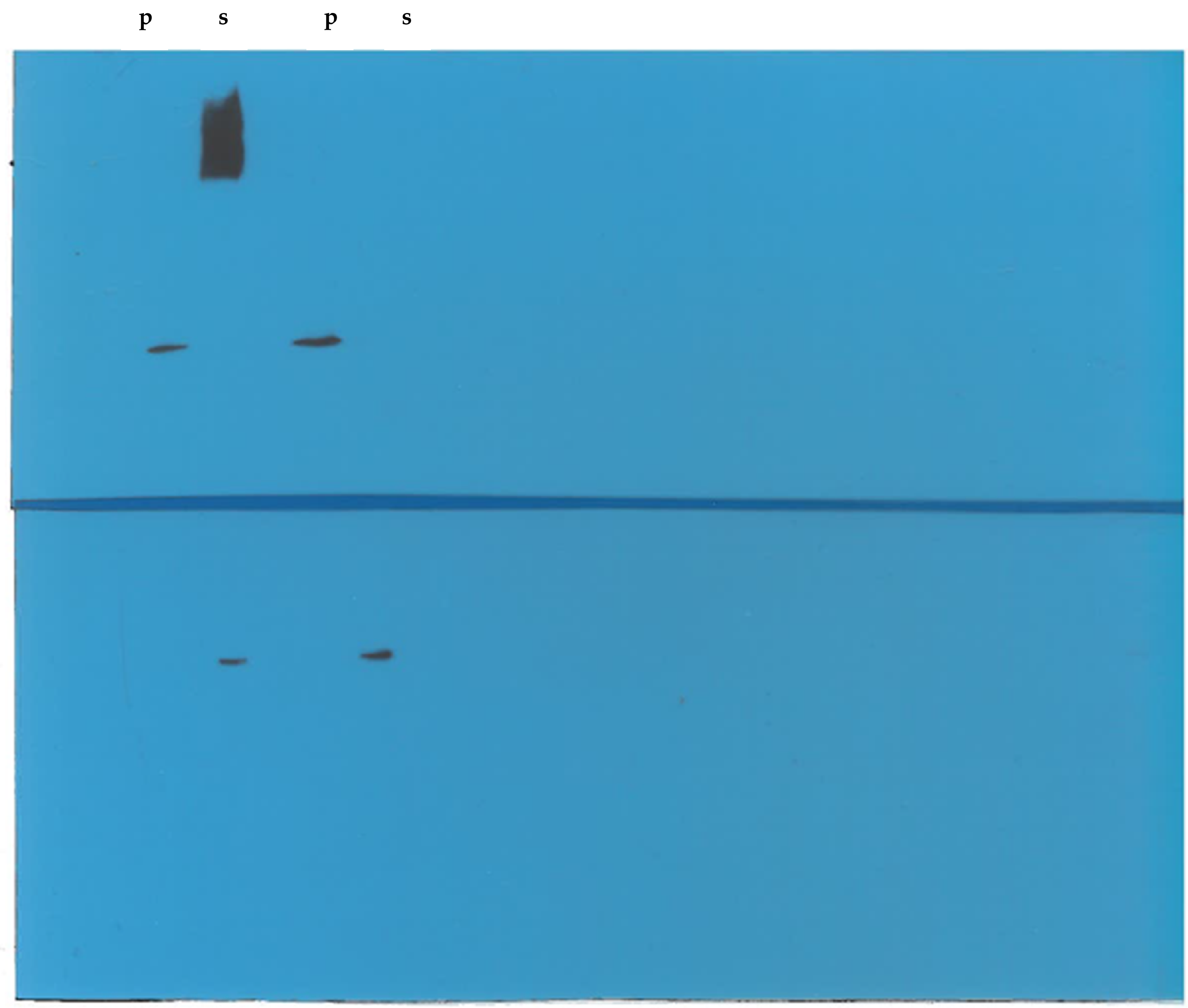

Figure S2. *Western blot of SAcP in LdWT and LdΔSAcP.* Uncropped western blot analysis of SAcP in *L. donovani* wild type (LdWT) and *L. donovani* mutant (ΔSAcP) strains. Cell lysates (p = parasite) and cell-culture supernatants (s = supernatants) were blotted using the α-SAcP primary rabbit antibody. The loading control was blotted with HSP83 (bottom).
